# Factors Affecting Platelet-Rich Plasma Success in Patients With Diabetic Foot Ulcer

**DOI:** 10.7759/cureus.40803

**Published:** 2023-06-22

**Authors:** Eyüp Murat Kanber, Harun Gulmez

**Affiliations:** 1 Department of Cardiovascular Surgery, Private Hospital, Istanbul, TUR; 2 Department of Cardiovascular Surgery, Private Gurlife Hospital, Istanbul, TUR

**Keywords:** glomerular filtration rate, smoking, prp, obesity, diabetes mellitus, diabetic foot ulcer

## Abstract

Introduction

Diabetic foot ulcers (DFU) are one of the most common complications of diabetes mellitus (DM). The use of platelet-rich plasma (PRP) in the treatment of DFU has been increasing in recent years. In the current study, we aimed to evaluate the factors affecting the success of PRP in the management of DFU.

Methods

The present study was planned in a prospective manner, and we enrolled patients with DFU in the study. Patients’ characteristics, DFU properties, and treatment outcomes were recorded. Patients with DFU were classified into two groups according to PRP success: healed DFU patients in Group 1 and non-healed DFU patients in Group 2. Groups were compared according to patient characteristics and treatment outcomes, and multivariate regression analysis was performed to clarify factors that predicted PRP success.

Results

In total, 182 patients were enrolled in the study, and DFU in 141 (77.5%) patients healed with PRP treatment. The mean body mass index (BMI) was significantly higher in the non-healed patients (p= 0.005). The smoking rate was 58.5% in the non-healed group and 39.7% in the healed group (p= 0.016). The duration of DM (17.9 years vs. 22.5 years, p= 0.003) was significantly longer, and patients with glomerular filtration rate (GFR) < 60 (44.0% vs. 63.4, p= 0.028) were significantly more common in the non-healed group. Multivariate regression analysis demonstrated that BMI ≥30 kg/m^2 ^and duration of DM ≥20 years were predictive factors for PRP failure in patients with DFU (p= 0.019 and p= 0.005). Smoking and GFR <60 were significantly associated with PRP failure (p= 0.040 and p= 0.044).

Conclusion

In our study, it was found that PRP was an effective treatment that improved DFU in 141 out of 182 patients. Moreover, the present study demonstrated for the first time that higher BMI, longer duration of DM, smoking, and lower GFR were significantly related to PRP failure in patients with DFU.

## Introduction

Diabetes mellitus (DM) is a chronic metabolic disease with abnormal increments in blood glucose levels. Due to dietary habits, including abnormal consumption of saturated fat and sugar, a more sedentary lifestyle, and increases in obesity incidence, the prevalence of DM is rapidly increasing, and research states that almost 11% of the world’s population will suffer from DM in 2050 [1]. Diabetic foot ulcers (DFU) are one of the most common complications of DM, and almost a quarter of patients are faced with DFU due to neuropathy, impaired immunity, infections, and chronic ischemia [2]. Previous studies emphasized that DFU was associated with deterioration in patient quality of life, increases in health care costs, extremity loss, and even death. Therefore, different treatment methods were described to treat DFU, including debridement and dressing, skin grafts, revascularization surgeries, and platelet-rich plasma (PRP) [3,4].

Platelet-rich plasma is obtained with the recombinant DNA technique, and the PRP solution includes cytokines, fibrin scaffolds, and growth factors that stimulate cell proliferation, cellular differentiation, and extracellular protein synthesis. Previous reports concluded that PRP is a safe, effective, and minimal invasive method for tissue healing [5]. Elsaid and colleagues compared PRP and saline dressing for the management of 24 patients with non-healing DFU, and the authors found that reduction of ulcer size was significantly better with PRP [6]. In another study, Alamdari et al. used conventional dressing along with silver sulfadiazine and PRP for DFU, and the healing rate was significantly higher in favor of the PRP group [7]. However, these studies did not focus on predictive factors for PRP success.

Although previous studies evaluated the effectiveness of PRP for the treatment of DFU, no studies to date have analyzed the parameters that affect PRP success. In the present study, our aim was to clarify factors that affect PRP success in the management of DFU.

## Materials and methods

The present study was planned in a prospective manner, and patients admitted to the cardiovascular surgery outpatient clinic between 2016 and 2021 at Private Gurlife Hospital, Istanbul, Turkey were evaluated. The research was carried out following the principles of the Declaration of Helsinki, and all patients provided their informed consent to take part in the study. Approval from the local ethics committee, with the identification number 2016-43, was obtained. Patients who had DFU were enrolled in the study. Before PRP application, all patients were informed about PRP's effects on tissue, possible complications, and success rate. All PRP procedures were performed by two cardiovascular specialists in the same manner. Patients who were ≤18 years old, patients with concomitant osteomyelitis, and patients with a history of chemotherapy and radiotherapy were excluded from the study. Other exclusion criteria were the presence of any hematological disease, having a low platelet count (<100,000/mm3), patients with irregular follow-up, and the presence of any vascular disease associated with chronic lower extremity ischemia.

Patient assessment

A detailed medical and surgical history was obtained from all patients, and a routine physical examination was performed to determine any ischemic signs, the presence of neuropathy, infective tissue, and the characteristics of ulcers. Patient age, gender, body mass index (BMI), presence of comorbidities, and duration of DM were recorded. Also evaluated were ulcer length and width, the status of ulcer edges and floor, presence of callus formation, and granulation tissue. Laboratory tests, including a complete blood count, glucose level, HbA1c level, glomerular filtration rate (GFR), liver function tests, and coagulation level, were performed. Also, radiography was performed to exclude osteomyelitis and skeletal deformities. In addition, Doppler sonography was done in patients with suspected vascular pathologies.

Preparation and application of PRP

**Figure 1:**
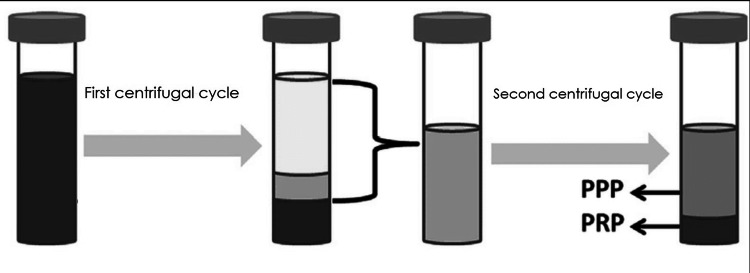
PRP preparation scheme PRP: platelet-rich plasma; PPP: platelet-poor plasma

PRP was prepared with 20 ml of the patients’ own blood. Blood was drawn from the venous system and placed into a tube containing citrate dextrose, and then two centrifugal cycles (3600 rounds/min and 2400 rounds/min) were performed. In the first centrifugal cycle, red blood cells (45% of all blood) were discarded, and platelet-poor plasma (PPP) and PRP (55% of all blood) were obtained for the second centrifugal cycle. Then, in the second centrifugal cycle, PPP (80% of the solution) was discarded and PRP (20% of the solution) was taken for use. The PRP preparation scheme is shown in Figure 1. Before the application of PRP, dead tissues and hyperkeratotic areas were debrided, and saline was used for wound irrigation. After PRP application, the wound was covered by vaseline gauze, sterile gauze, and a non-compressive bandage, respectively. The cycles were performed twice a week, and the protocol continued for 24 weeks if required. Success was defined as complete ulcer healing and ≥ 50% reduction in wound size at three months post-procedure.

**Figure 2:**
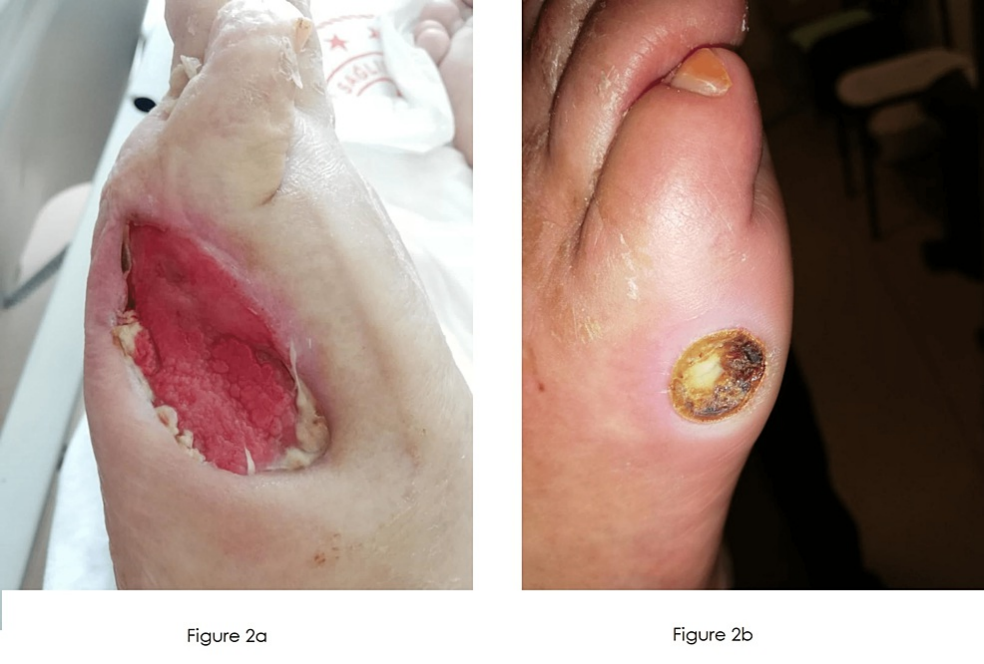
Example pictures of patients (Figure 2a: healed, Figure 2b: non-healed)

Finally, patients with DFU were classified into two groups according to PRP success: healed patients in Group 1 and non-healed patients in Group 2. Groups were compared according to patient characteristics, and multivariate regression analysis was performed to clarify factors that predicted PRP success in patients with DFU. Example pictures of healed and non-healed patients are shown in Figure 2.

Statistical analysis

IBM SPSS Statistics for Windows, Version 25.0 (released 2017; IBM Corp., Armonk, New York, United States) was used. The normality of parameter distributions was checked by skewness and kurtosis tests. The student t-test was used for the comparison of variables between the groups. Descriptive data are shown as the mean and standard deviation. Categorical variables were compared using the χ2 test or Fisher’s exact test. Multivariate analysis was applied to parameters that were significant in univariate analysis to evaluate the factors affecting treatment success. The data were analyzed at the 95% confidence level, and a p value of less than 0.05 was accepted as statistically significant.

## Results

In total, 182 patients were enrolled in the study, and DFU in 141 (77.5%) patients healed with PRP treatment. The mean age of 182 patients was 62.9 years, and 94 (51.6%) patients were male. The BMI of the study population was 28.4 kg/m2. Hypertension, coronary artery disease, and hyperlipidemia were detected in 70 (38.5%) patients, 67 (36.8%) patients, and 74 (40.6%) patients, respectively. The smoking rate was 43.9%. The mean duration of DM was 18.9 years, and the mean HbA1C level was 7.5. Also, the mean wound size was 4.3 cm, and 88 (48.3%) patients had a GFR level <60 (Table 1).

**Table 1:** Data for all patients *mean ± standard deviation BMI: body mass index; HbA1c: hemoglobin A1c; GFR: glomerular filtration rate

	n:182
Age (years)*	62.9±9.8
Sex, n (%)	
Male	94 (51.6%)
Female	88 (48.4%)
BMI (kg/m²)*	28.4±5.1
Hypertension, n (%)	70 (38.5%)
Coronary artery disease, n (%)	67 (36.8%)
Hyperlipidemia, n (%)	74 (40.6%)
Smoking, n (%)	80 (43.9%)
Alcohol use, n (%)	70 (38.5%)
HbA1C (%)*	7.5±1.2
Duration of diabetes mellitus (years)*	18.9±8.9
Mobility limited, n (%)	89 (48.9%)
Wound size (cm)*	4.3±1.6
Platelet count (10^3 ^uL)*	250.3±59.3
GFR (mL/min/1.73 m^2^) , n (%)	
≤ 60	88 (48.3%)
> 60	94 (51.7%)
Ankle-brachial index*	0.7±0.2

Comparison of healed and non-healed patients with DFU revealed that age, gender, presence of hypertension, presence of coronary artery disease, presence of hyperlipidemia, and HbA1c level were comparable between the groups (p= 0.502, p= 0.950, p= 0.169, p= 0.973, p= 0.809, and p= 0.621, respectively). However, mean BMI was significantly higher in the non-healed patients (27.8 kg/m2 vs. 30.3 kg/m2, p= 0.005). In addition, the smoking rate was 58.5% in the non-healed group and 39.7% in the healed group (p= 0.016). Moreover, the duration of DM (17.9 years vs. 22.5 years, p= 0.003) was significantly longer, and patients with GFR < 60 (44.0% vs. 63.4, p= 0.028) were significantly more common in the non-healed group. Comparisons between the two groups are summarized in Table 2.

**Table 2:** Comparison of data according to treatment success status *mean ± standard deviation BMI: body mass index; HbA1c: hemoglobin A1c; GFR: glomerular filtration rate

	Healed group (n:141)	Non-healed group (n:41)	P value
Age (years)*	63.2±9.6	62.0±10.8	0.502
Sex, n (%)			0.950
Male	73 (51.8%)	21 (51.2%)	
Female	68 (48.2%)	20 (48.8%)	
BMI (kg/m²)*	27.8±5.0	30.3±5.1	0.005
Hypertension, n (%)	58 (41.1%)	12 (29.3%)	0.169
Coronary artery disease, n (%)	52 (36.9%)	15 (36.6%)	0.973
Hyperlipidemia, n (%)	58 (41.1%)	16 (39.0%)	0.809
Smoking, n (%)	56 (39.7%)	24 (58.5%)	0.016
Alcohol use, n (%)	55 (39.0% )	15 (36.6%)	0.779
HbA1c (%)*	7.5±1.2	7.6±1.2	0.621
Duration of diabetes mellitus (years)*	17.9±8.9	22.5±7.8	0.003
Mobility limited, n (%)	67 (47.5%)	22 (53.7%)	0.489
Wound size (cm)*	4.3±1.6	4.5±1.6	0.403
Platelet count (10^3 ^uL)*	249.0±60.4	254.7±55.9	0.587
GFR (mL/min/1.73 m^2^), n (%)			0.028
≤ 60	62 (44.0%)	26 (63.4%)	
> 60	79 (56.0%)	15 (36.6%)	
Ankle-brachial index*	0.7±0.2	0.7±0.2	0.148

Multivariate regression analysis demonstrated that BMI ≥30 kg/m2 and duration of DM ≥20 years were predictive factors for PRP failure in patients with DFU (p= 0.019 and p= 0.005). Moreover, smoking increased the PRP failure risk by 2.216 times (p= 0.040). Lastly, GFR <60 % was significantly associated with PRP failure in comparison with GFR >60 in patients with DFU (odds ratio 2.198, p= 0.044). The outcomes of multivariate regression analysis to determine predictive factors for PRP in patients with DFU are presented in Table 3.

**Table 3:** Multivariate analysis of factors affecting treatment success CI: confidence interval

	Odds ratio	95% CI	P value
Body mass index (>30 kg/m2 / ≤30 kg/m2)	2.465	1.160-5.240	0.019
Time of diabetes mellitus (>20 yeaars / ≤20 years)	3.082	1.416-6.707	0.005
Smoking status (Yes/No)	2.216	1.037-4.739	0.040
Glomerular filtration rate (≤60 % / >60 %)	2.198	1.022-4.724	0.044

## Discussion

The management of complications related to DM necessitates meticulous and intricate treatment approaches. DFU is a very common complication of DM, and many different treatment methods have been defined for the management of DFU. PRP is a relatively new technique, and we believe that it is crucial to identify predictive factors that may impact PRP success. Also, revealing parameters about PRP success will enable the correction of risk factors and increase the success of PRP. In the present study, we compared healed and non-healed patients with DFU after PRP, and we found that higher BMI, longer duration of DM, smoking, and lower GFR were predictive factors for PRP failure in patients with DFU.

The effect of obesity on wound healing is one of the most interesting topics. Insufficient penetration of antibiotics, inadequate oxygenation of tissue, and impaired immunity may play roles in poor wound healing in obese patients [8]. Patel and colleagues investigated the effect of obesity on lower extremity arterial revascularization, and the authors found that obesity did not affect graft patency or survival rate but increased preoperative wound infection [9]. Also, Lavery et al. stated that obesity significantly increased peak pressure in the heel region, first metatarsal, and lesser metatarsal in diabetic patients, which could be associated with DFU [10]. In the present study, we found a significant correlation between BMI >30 kg/m2 and poor DFU healing.

Long-term DM plays a role in genetic, neurological, and nephrological pathologies. Vigorita et al. emphasized that DM itself, but not DM duration, was significantly associated with atherosclerosis and tissue ischemia [11]. In contrast, Oguejiofor et al. stated that DM duration longer than >15 years was significantly associated with peripheral neuropathy and DFU [12]. In the present study, DM duration >20 years was found to be a predictive factor for DFU. However, we did not use objective tests for the evaluation of neuropathy and atherosclerosis, which may be subjects for other studies.

Previous studies stated that smoking is a risk factor for delayed and poor wound healing due to tissue hypoxia, deteriorated collagen synthesis, and thrombotic occlusion. Jones and colleagues investigated the role of smoking in 1789 patients with lower extremity bypass surgery outcomes and concluded that the graft patency rate and overall survival were significantly worse in patients who actively smoked [13]. A review performed by Xia et al. supported the view that diabetic patients should stop smoking to prevent DFU and improve the prognosis for DFU [14]. In the present study, we found a negative correlation between smoking and PRP success. Thus, we support the idea that all diabetic patients who will undergo PRP treatment for DFU should quit smoking.

Kidney dysfunction results in poor wound healing due to uremic toxins, impaired blood circulation, and loss of proteins. Heller and colleagues investigated the effect of chronic kidney disease on abdominal surgery, and the authors found a significant correlation between chronic kidney disease and incisional hernia [15]. In another study, Zubair et al. analyzed patients with DFU, and poor wound healing and amputation occurred in patients with GFR <30 [16]. In the present study, we found that GFR <60 was associated with poor DFU healing.

The present study has some limitations. First of all, the present study focused on the short-term results of PRP without examining long-term outcomes. Secondly, we did not evaluate which treatments were performed in cases of PRP failure, which may be the subject of further studies. Lastly, we did not perform a cost analysis of PRP, which could be clarified in further studies.

## Conclusions

Our study found that PRP is an effective treatment that healed DFU in four out of five patients. It is critical to choose the right treatment for DFU and to predict the success of the treatment to be applied. The present study demonstrated for the first time that higher BMI, longer duration of DM, smoking, and lower GFR were significantly related to PRP failure in patients with DFU.

## References

[1] Zimmet P, Alberti KG, Magliano DJ, Bennett PH (2016). Diabetes mellitus statistics on prevalence and mortality: facts and fallacies. Nat Rev Endocrinol.

[2] Russo S, Landi S, Courric S (2022). Cost-effectiveness analysis for the treatment of diabetic foot ulcer in France: platelet-rich plasma vs standard of care. Clinicoecon Outcomes Res.

[3] Kirsner RS, Delhougne G, Searle RJ (2020). A cost-effectiveness analysis comparing single-use and traditional negative pressure wound therapy to treat chronic venous and diabetic foot ulcers. Wound Manag Prev.

[4] Linertová R, Del Pino-Sedeño T, Pérez LG (2021). Cost-effectiveness of platelet-rich plasma for diabetic foot ulcer in Spain. Int J Low Extrem Wounds.

[5] Deng J, Yang M, Zhang X, Zhang H (2023). Efficacy and safety of autologous platelet-rich plasma for diabetic foot ulcer healing: a systematic review and meta-analysis of randomized controlled trials. J Orthop Surg Res.

[6] Elsaid A, El-Said M, Emile S, Youssef M, Khafagy W, Elshobaky A (2020). Randomized controlled trial on autologous platelet-rich plasma versus saline dressing in treatment of non-healing diabetic foot ulcers. World J Surg.

[7] Malekpour Alamdari N, Shafiee A, Mirmohseni A, Besharat S (2021). Evaluation of the efficacy of platelet-rich plasma on healing of clean diabetic foot ulcers: a randomized clinical trial in Tehran, Iran. Diabetes Metab Syndr.

[8] Guo S, Dipietro LA (2010). Factors affecting wound healing. J Dent Res.

[9] Patel VI, Hamdan AD, Schermerhorn ML (2007). Lower extremity arterial revascularization in obese patients. J Vasc Surg.

[10] Lavery LA, Armstrong DG, Vela SA, Quebedeaux TL, Fleischli JG (1998). Practical criteria for screening patients at high risk for diabetic foot ulceration. Arch Intern Med.

[11] Vigorita VJ, Moore GW, Hutchins GM (1980). Absence of correlation between coronary arteria atherosclerosis and severity or duration of diabetes mellitus of adult onset. The American Journal of Cardiology.

[12] Oguejiofor OC, Odenigbo CU, Oguejiofor CB (2010). Evaluation of the effect of duration of diabetes mellitus on peripheral neuropathy using the United Kingdom screening test scoring system, bio-thesiometry and aesthesiometry. Niger J Clin Pract.

[13] Jones DW, Goodney PP, Eldrup-Jorgensen J (2018). Active smoking in claudicants undergoing lower extremity bypass predicts decreased graft patency and worse overall survival. J Vasc Surg.

[14] Xia N, Morteza A, Yang F, Cao H, Wang A (2019). Review of the role of cigarette smoking in diabetic foot. J Diabetes Investig.

[15] Heller A, Westphal SE, Bartsch P, Haase M, Mertens PR (2014). Chronic kidney disease is associated with high abdominal incisional hernia rates and wound healing disturbances. Int Urol Nephrol.

[16] Zubair M, Malik A, Ahmad J (2011). The impact of creatinine clearance on the outcome of diabetic foot ulcers in north Indian tertiary care hospital. Diabetes Metab Syndr.

